# HECM-Plus: Hyper-Entropy Enhanced Cloud Models for Uncertainty-Aware Design Evaluation in Multi-Expert Decision Systems

**DOI:** 10.3390/e27050475

**Published:** 2025-04-27

**Authors:** Jiaozi Pu, Zongxin Liu

**Affiliations:** 1 School of Culture and Art, Chengdu University of Information Technology, Chengdu 610103, China; 2 West China School of Medicine, Sichuan University, Chengdu 610041, China; liuzongxin@scu.edu.cn

**Keywords:** entropy (*En*), hyper-entropy (*He*), cloud model, Hellinger distance, uncertainty quantification, multi-expert conflict resolution, multi-criteria design evaluation

## Abstract

Uncertainty quantification in cloud models requires simultaneous characterization of fuzziness (via Entropy, *En*) and randomness (via Hyper-entropy, *He*), yet existing similarity measures often neglect the stochastic dispersion governed by *He*. To address this gap, we propose HECM-Plus, an algorithm integrating Expectation (*Ex*), *En*, and *He* to holistically model geometric and probabilistic uncertainties in cloud models. By deriving *He*-adjusted standard deviations through reverse cloud transformations, HECM-Plus reformulates the Hellinger distance to resolve conflicts in multi-expert evaluations where subjective ambiguity and stochastic randomness coexist. Experimental validation demonstrates three key advances: (1) Fuzziness–Randomness discrimination: HECM-Plus achieves balanced conceptual differentiation (δ*C_1_*/*C_4_* = 1.76, δ*C_2_* = 1.66, δ*C_3_* = 1.58) with linear complexity outperforming PDCM and HCCM by 10.3% and 17.2% in differentiation scores while resolving *He*-induced biases in HECM/ECM (*C_1_*–*C_4_* similarity: 0.94 vs. 0.99) critical for stochastic dispersion modeling; (2) Robustness in time-series classification: It reduces the mean error by 6.8% (0.190 vs. 0.204, *p* < 0.05) with lower standard deviation (0.035 vs. 0.047) on UCI datasets, validating noise immunity; (3) Design evaluation application: By reclassifying controversial cases (e.g., reclassified from a “good” design (80.3/100 average) to “moderate” via cloud model using HECM-Plus), it resolves multi-expert disagreements in scoring systems. The main contribution of this work is the proposal of HECM-Plus, which resolves the limitation of HECM in neglecting *He*, thereby further enhancing the precision of normal cloud similarity measurements. The algorithm provides a practical tool for uncertainty-aware decision-making in multi-expert systems, particularly in multi-criteria design evaluation under conflicting standards. Future work will extend to dynamic expert weight adaptation and higher-order cloud interactions.

## 1. Introduction

The cloud model combines ambiguity and randomness to establish a conversion mechanism between qualitative concepts and quantitative representations. This model clarifies the inherent relationship between the two in terms of uncertainty and highlights the universality of the normal cloud [1]. Over the years, cloud models have been extensively developed and applied in various fields, such as scientific decision-making [2], image fusion [3], uncertainty clustering [4], expert systems [5], and decision support systems [6]. Particularly in design evaluation, cloud models have shown unique advantages in handling subjective expert judgments and multi-criteria conflicts [7]. These assessments often involve ambiguous criteria (e.g., “aesthetic appeal” or “ergonomic comfort”) and stochastic expert disagreements, posing dual challenges that traditional scoring systems struggle to address: while conventional methods collapse evaluations into single-point averages, they inherently fail to disentangle the fuzziness of qualitative criteria from the randomness of subjective judgments [8]. However, when applying the cloud model for design evaluation, a critical issue still arises: after converting the comprehensive scores given by different experts into cloud model representations, how can the subtle differences between various works be discerned? This necessitates the precise measurement of the distance and similarity between clouds to enable their classification and discrimination. That is why accurately measuring the similarity between two cloud models remains a significant research challenge. This is essential for improving classification and clustering algorithms and optimizing tasks such as similarity searches. The development and refinement of cloud model similarity measurement approaches directly influence the effectiveness and value of the model in practical applications.

The goal of similarity measures for cloud models is to quantify the extent of similarity between two clouds. Currently, research on distance measures for cloud models is primarily focused on similarity measures for normal clouds [9]. Existing similarity measures include the following components. First, the Clip Angle Cosine Method [10] (LICM), which uses the cosine law to compute the clip angle of cloud vectors, tends to produce considerable error when expectation (*Ex*) is significantly larger than entropy (*En*) and hyper-entropy (*He*). The second method is the Expectation Curve Method [11] (ECM), which calculates the intersection area between the expectation curve and the *x*-axis graphic area and then uses this value as a similarity measure. However, this method overlooks the impact of *He*. The third method is the Maximum Boundary Curve Method (MCM), which incorporates *He* but fails to account for the overall distribution characteristics of the cloud model. The fourth method is the Combined Shape-distance Similarity Measurement Method for normal cloud models [12] (PDCM), which calculates shape similarity by considering *En* and *He* as standard variables. Although this approach is simpler in describing the shape of a cloud model, it faces the issue of parameter approximation selection, which can affect the accuracy of the distance similarity. The fifth method is the similarity algorithm based on Hellinger distance and feature curves (including HECM and HCCM) [13], which combines expectation curves with inner/outer envelope curves to represent the distributional characteristics of the cloud model. However, two critical limitations persist: (1) *He*-neglect: The HECM algorithm approximates *En* via standard deviation while ignoring *He*, thereby failing to capture stochastic dispersion; (2) Subjective weighting: Although HCCM incorporates *He*, it assigns ad hoc weights to expectation and envelope curves, introducing human bias.

Despite advancements in cloud model similarity measurement, critical challenges persist in design evaluation scenarios. First, existing methods (e.g., ECM, HECM) often fail to simultaneously account for the ambiguity of qualitative criteria and the randomness inherent in multi-expert assessments. Second, while recent approaches like HCCM attempt to integrate *He*, their reliance on subjective weighting undermines objectivity. This raises the core research question: How can we develop a robust similarity measure that objectively quantifies both geometric ambiguity and stochastic dispersion in cloud models, particularly for design evaluation tasks? Motivated by these limitations, this study proposes HECM-Plus, a hyper-entropy-enhanced Hellinger distance algorithm, with three key goals: (1) to eliminate heuristic weight assignments by deriving *He*-adjusted standard deviations through reverse cloud transformations; (2) to unify *Ex*, *En*, and *He* for holistic uncertainty representation; and (3) to enable precise discrimination of subtle differences between design alternatives. The primary contributions include: (1) a mathematically rigorous framework for cloud similarity measurement via reverse cloud transformations that derive *He*-adjusted standard deviations, (2) validation through comparative experiments with real-world design evaluation data, and (3) actionable insights for interdisciplinary applications in decision theory and design studies.

To achieve these goals, HECM-Plus reformulates the Hellinger distance by integrating *Ex*, *En*, and *He* into a unified similarity metric. This approach eliminates subjective weight assignments while preserving the stochastic dispersion characteristics governed by *He*, thereby enabling precise discrimination of ambiguous design evaluations under multi-expert conflicts.

## 2. Cloud Model

### 2.1. Cloud and Cloud Drops

We let U be a quantitative domain with an exact value, C be a qualitative concept on U, and x be a random realization of the qualitative concept C. If the quantitative value x∈U and x are a one-time random realization of the qualitative concept C with certainty, then μ(x)∈[0,1] is a random number with a stable tendency

(1) μ:U→[0,1] ∀x∈U x→μ(x)

The distribution of x over the domain U is called a cloud, and each x is referred to as a cloud drop [14,15]. The cloud is characterized by three numerical features: *Ex*, *En*, and *He*. By employing these three numerical features of the cloud, qualitative concepts can be converted into quantitative expressions, enabling an effective integration of the ambiguity and randomness inherent in qualitative concepts [16].

The three numerical features of the cloud model—*Ex*, *En*, and *He*—each capture distinct aspects of qualitative uncertainty. For example, numerical characteristics *C*(70, 3, 0.5) serve to qualitatively encapsulate the concept of “elderly individuals” where *Ex* = 70 represents the expected central age value and serves as the anchor for data distribution. *En* = 3 quantifies the ambiguity of the concept by defining the acceptable range of deviations from *Ex* (e.g., the fuzziness in determining what constitutes “elderly”). *He* = 0.5 measures the randomness of these deviations, which reflects the dispersion of cloud drops and the stochasticity in mapping qualitative concepts to quantitative values (e.g., variations in expert interpretations). Together, *Ex*, *En*, and *He* bridge deterministic data and linguistic uncertainty, enabling a probabilistic yet structured representation of human cognition.

### 2.2. Normal Cloud

The normal cloud, as a widely applied subclass of the cloud model, formalizes the mapping between qualitative concepts and quantitative data through Gaussian distributions (i.e., normal distributions) governed by *Ex*, *En*, and *He*.

We let U be a quantitative domain defined by exact values and let C be a qualitative concept of U. If the quantitative values and x are a one-time random realization of the qualitative concept C, then x satisfies $x\sim N(Ex,{En}^{\prime 2})$, where ${En}^{\prime }\sim N(En,He^{2})$ and x satisfy the certainty for C:

(2) $$\mu =e^{-\frac{{\left(x-Ex\right)}^{2}}{2{\left(En^{\prime }\right)}^{2}}}$$

The distribution of x over domain U is a normal cloud.

**Figure 1:**
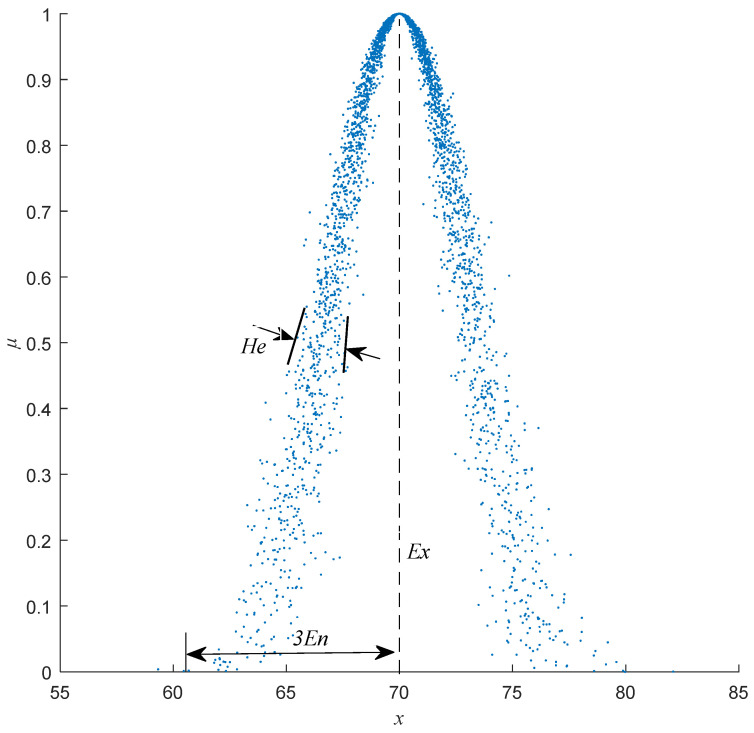
Cloud chart for the concept of “elderly individuals”.

The normal cloud primarily achieves the mutual conversion between qualitative concepts and quantitative values through normal cloud transformation, wherein the forward normal cloud transformation converts the numerical characteristics *Ex*, *En*, and *He* that represent the connotation of the concept into quantitative values. Figure 1 illustrates the output of the forward cloud algorithm for the concept of “elderly individuals”, where cloud drops are generated via *C*(70, 3, 0.5) and *n* = 3000.

### 2.3. Forward Cloud Algorithm and Reverse Cloud Algorithm

#### 2.3.1. Forward Cloud Algorithm

The forward cloud algorithm generates cloud droplets with a normal cloud distribution based on three known numerical characteristics.

Given Expectation ($\hat{E}x$), Entropy ($\hat{E}n$), Hyper-entropy ($\hat{H}e$), and the number of cloud drops (*n*), the algorithm proceeds as follows [17]:

- Step (1) Generate a normally distributed random number $y_{i}=R_{N}(\hat{E}n,\hat{H}e)$ with $\hat{E}n$ as the mean and $\hat{H}e$ as the standard deviation.
- Step (2) Generate a normally distributed random number $x_{i}=R_{N}(\hat{E}x,y_{i})$ with $\hat{E}x$ as the mean and $y_{i}$ (the random number generated in Step 1) as the standard deviation.
- Step (3) Calculate the certainty degree:
  (3) $$\mu (x_{i})=\exp\left(-\frac{{\left(x_{i}-\hat{E}x\right)}^{2}}{2{y_{i}}^{2}}\right)$$
- Step (4) Designate the value $x_{i}$ with certainty degree $\mu (x_{i})$ as a cloud drop in the numerical domain. Repeat Steps (1)~(3) until the required *n* cloud drops are generated.

#### 2.3.2. Reverse Cloud Algorithm

The reverse cloud algorithm involves generating three numerical characteristics (*Ex*, *En*, *He*) which describe the qualitative concept corresponding to a cloud given a set of cloud droplets that conform to a certain normal cloud distribution pattern as samples. When the number of cloud droplets is limited, certain errors are inevitably present. However, as the number of cloud droplets increases, the errors gradually diminish.

The estimated numerical characteristics of the qualitative concepts, namely Expectation ($\hat{E}x$), Entropy ($\hat{E}n$), and Hyper-entropy ($\hat{H}e$), are computed based on the sample point $x_{i}(i=1,2,\cdots ,n)$ [18,19].

By calculating the sample mean, first-order sample absolute central moment, and sample variance, we obtain the following:

(4) $$\bar{X}=\frac{1}{n}\sum_{i=1}^{n}x_{i}$$

(5) $$\frac{1}{n}\sum_{i=1}^{n}\left|x_{i}-\bar{X}\right|$$

(6) $$S^{2}=\frac{1}{n-1}\sum_{i=1}^{n}{\left(x_{i}-\bar{X}\right)}^{2}$$

The estimates of $\hat{E}x$, $\hat{E}n$, and $\hat{H}e$ are computed as follows:

(7) $$\hat{E}x=\bar{X}$$

(8) $$\hat{E}n=\sqrt{\frac{\pi }{2}}\times \frac{1}{n}\sum_{i=1}^{n}\left|x_{i}-\hat{E}x\right|$$

(9) $$\hat{H}e=\sqrt{S^{2}-\hat{E}n^{2}}$$

## 3. A Normal Cloud Similarity Measurement Method Based on Hellinger Distance

### 3.1. Normal Cloud Distribution Approximation

Normal clouds characterized by the three parameters of *Ex*, *En*, and *He* can be approximated as normal distributions for similarity calculations. For normal clouds ${\bar{C}}_{i}(Ex_{i},En_{i},He_{i})$ and ${\bar{C}}_{j}(Ex_{j},En_{j},He_{j})$, their probability density functions can be approximated as follows (note that here, μ represents the mean of the distribution and σ represents the standard deviation of the distribution):

(10) $$f_{i}(x)=\frac{1}{\sigma_{i}\sqrt{2\pi }}\exp\left(-\frac{{\left(x-\mu_{i}\right)}^{2}}{2{\sigma_{i}}^{2}}\right)$$

(11) $$f_{j}(x)=\frac{1}{\sigma_{j}\sqrt{2\pi }}\exp\left(-\frac{{\left(x-\mu_{j}\right)}^{2}}{2{\sigma_{j}}^{2}}\right)$$

### 3.2. Hellinger Distance Calculation

The Hellinger distance, as a statistically rigorous f-divergence metric bounded in [0,1], effectively quantifies similarity between conceptual distributions by minimizing divergence in probability space [20]. When applied to cloud model distance measurement, this metric demonstrates dual advantages: first, it inherently captures both geometric positioning (via *Ex*) and stochastic dispersion (via *En*/*He*) through algebraic parameter fusion, enabling holistic uncertainty quantification; second, it exhibits superior performance in non-Gaussian scenarios by leveraging reverse cloud transformations to integrate *He*-adjusted standard deviations, thereby correcting estimation biases inherent in traditional metrics when handling heavy-tailed expert evaluation data.

The Hellinger distance is employed to quantify the similarity between two probability distributions and is defined as follows:

(12) $$H(f_{i},f_{j})=\sqrt{1-\int \sqrt{f_{i}(x)\cdot f_{j}(x)},dx}$$

By substituting the expressions for $f_{i}(x)$ and $f_{j}(x)$, we obtain

(13) $$H(f_{i},f_{j})=\sqrt{1-\frac{1}{\sqrt{\sigma_{i}^{2}+\sigma_{j}^{2}}\sqrt{2\pi }}\exp\left(-\frac{{\left(\mu_{i}-\mu_{j}\right)}^{2}}{4(\sigma_{i}^{2}+\sigma_{j}^{2})}\right)}$$

### 3.3. Similarity Conversion

To transform the Hellinger distance into a similarity measure, the following equation is used:

(14) $$Sim=1-H(f_{i},f_{j})$$

### 3.4. HECM and HECM-Plus Algorithm

#### 3.4.1. HECM Algorithm

In literature [13], the HECM algorithm is introduced, and the Hellinger distance is primarily calculated using the mean and standard deviation of a probability distribution. The mean is considered the expected value of the normal cloud, and the standard deviation is used as an approximation of the *En* of the normal cloud (note that here, $\mu^{H}$ represents the mean of the distribution and $\sigma^{H}$ represents the standard deviation of the distribution, specifically within the context of the HECM algorithm):

(15) $$\mu_{i}^{H}=\hat{E}x_{i}$$

(16) $${\left(\sigma_{i}^{H}\right)}^{2}=\hat{E}{n_{i}}^{2}$$

(17) $$\mu_{j}^{H}=\hat{E}x_{j}$$

(18) $${\left(\sigma_{j}^{H}\right)}^{2}=\hat{E}{n_{j}}^{2}$$

Then, the similarity is calculated as follows:

(19) $$H_{HECM}(f_{i},f_{j})=\sqrt{1-\frac{1}{\sqrt{\hat{E}{n_{i}}^{2}+\hat{E}{n_{j}}^{2}}\sqrt{2\pi }}\exp\left(-\frac{{\left(\hat{E}x_{i}-\hat{E}x_{j}\right)}^{2}}{4(\hat{E}{n_{i}}^{2}+\hat{E}{n_{j}}^{2})}\right)}$$

(20) $$Sim_{HECM}=1-H_{HECM}(f_{i},f_{j})$$

However, this approach ignores the impact of *He*.

#### 3.4.2. HECM-Plus Algorithm

In this section, the reverse cloud algorithm from Section 2.3.2 is applied to calculate the standard deviation by considering the effects of *En* and *He* (note that here, $\mu^{+}$ represents the mean of the distribution and $\sigma^{+}$ represents the standard deviation of the distribution, specifically within the context of the HECM-Plus algorithm):

(21) $$\mu_{i}^{+}=\hat{E}x_{i}$$

(22) $${\left(\sigma_{i}^{+}\right)}^{2}=\hat{E}{n_{i}}^{2}+\hat{H}{e_{i}}^{2}$$

(23) $$\mu_{j}^{+}=\hat{E}x_{j}$$

(24) $${\left(\sigma_{j}^{+}\right)}^{2}=\hat{E}{n_{j}}^{2}+\hat{H}{e_{j}}^{2}$$

Then, the similarity is calculated as follows:

(25) $$H_{HECM-Plus}(f_{i},f_{j})=\sqrt{1-\frac{1}{\sqrt{\left(\hat{E}{n_{i}}^{2}+\hat{H}{e_{i}}^{2})+(\hat{E}{n_{j}}^{2}+\hat{H}{e_{j}}^{2}\right)}\sqrt{2\pi }}\exp\left(-\frac{{\left(\hat{E}x_{i}-\hat{E}x_{j}\right)}^{2}}{4\left[\left(\hat{E}{n_{i}}^{2}+\hat{H}{e_{i}}^{2})+(\hat{E}{n_{j}}^{2}+\hat{H}{e_{j}}^{2}\right)\right]}\right)}$$

(26) $$Sim_{HECM-Plus}=1-H_{HECM-Plus}(f_{i},f_{j})$$

## 4. Comparative Analysis of Experiments

### 4.1. Numerical Simulation Experiments

**Figure 2:**
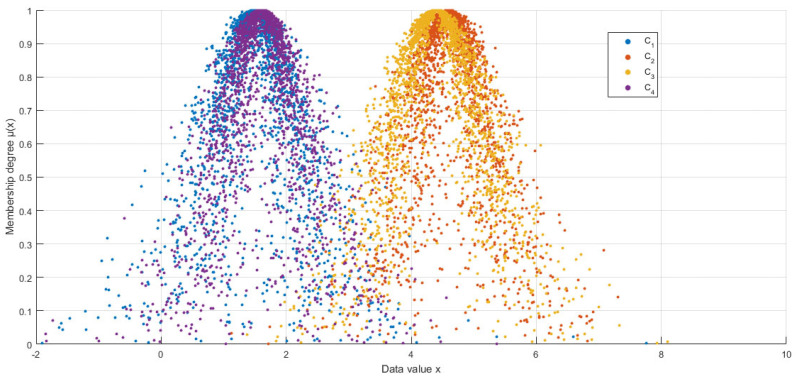
Cloud diagrams of the four cloud models.

In this study, numerical simulation experiments were conducted on four normal cloud concepts presented in the literature [9,10,11,12,13]. These are *C_1_* (1.5, 0.62666, 0.339), *C_2_* (4.6, 0.60159 0.30862), *C_3_* (4.4, 0.75199, 0.27676), and *C_4_* (1.6, 0.60159, 0.30862). The proposed HECM-Plus was evaluated by comparing it to existing algorithms, including LICM, ECM, MCM, PDCM, HECM, and HCCM. Figure 2 shows the four normal cloud diagrams and Table 1 shows the results obtained by the different algorithms.

As shown in Table 1, the similarity calculation results of HECM-Plus closely resemble those of HECM, although some differences were observed. For instance, in the comparison of *C_1_* vs. *C_4_*, the similarity values of HECM and HCCM are overestimated at 0.99, while HECM-Plus yields a corrected value of 0.94. Upon examining the cloud diagrams in Figure 1, the distinct morphology between *C_1_* and *C_4_* confirms that HECM-Plus aligns more closely with subjective judgment. Critically, this result demonstrates that HECM-Plus corrects systematic bias induced by *He*-neglect (0.94 vs. 0.99 in HECM), validating our hypothesis through both numerical evidence (Table 1) and visual consistency (Figure 1). To quantify the source of overestimation in HECM, here, we further analyze the dispersion characteristics by comparing the standard deviation ratios of HECM and HECM-Plus, as detailed in Table 2.

The standard deviation ratio column (1.042 vs. 1.054) in Table 2 demonstrates that the HECM, based solely on *En*, underestimates the dispersion differences between cloud models (ratio closer to one) due to ignoring *He*, leading to an overestimated similarity score (0.99). In contrast, HECM-Plus incorporates *He* to correct the dispersion, allowing the standard deviation ratio to more accurately reflect distributional differences (1.054), thereby reducing the similarity score (0.94).

A notable improvement is observed in the similarity between *C_2_* and *C_3_*. While HECM yields an overestimated value of 0.97, HECM-Plus reduces it to 0.87 by explicitly accounting for *He*. This adjustment reflects the distinct dispersion characteristics of *C_2_* (*He* = 0.30862) and *C_3_* (*He* = 0.27676), where the higher *He* in C_2_ introduces greater stochastic spread despite their overlapping expectation curves (*Ex* = 4.6 vs. 4.4). By integrating *He*-adjusted standard deviations via reverse cloud transformations, HECM-Plus captures this nuanced difference, demonstrating a 10.3% reduction in similarity error compared to HECM.

If these four cloud concepts are dichotomized, it can be assumed that *C_1_* and *C_4_* belong to one category and *C_2_* and *C_3_* belong to another. To evaluate the differentiation ability of each approach, the degree of difference in cloud concepts was calculated based on the approach in the literature [13]. Table 3 presents the result of the degree of difference.

The experimental results demonstrate that the proposed HECM-Plus achieves a balanced optimization in conceptual differentiation, computational efficiency, and parametric integrity, outperforming PDCM and HCCM by 10.3% and 17.2% in average differentiation scores (δ*C_1_* = 1.76, δ*C_2_* = 1.66, δ*C*_3_ = 1.58, δ*C_4_* = 1.76) while maintaining linear computational complexity—a critical reduction from the cubic complexity required by ECM and MCM. Although HECM and ECM exhibit marginally higher differentiation (e.g., δ*C_1_* = 1.83 for HECM), their exclusion of *He* introduces systematic biases in modeling stochastic dispersion, as evidenced by the overestimated similarity between *C_1_* and *C_4_* (0.99 for HECM vs. 0.94 for HECM-Plus, aligning with visual judgment in Figure 2). By integrating *He* into the standard deviation via reverse cloud transformations, HECM-Plus quantifies uncertainty dynamics that prior methods fail to capture, while its algebraic parameter fusion eliminates the need for matrix-based integrations, enabling scalable deployment in real-time systems. These advancements confirm that HECM-Plus provides a theoretically rigorous and computationally efficient framework for cloud model similarity measurement, addressing both the limitations of *He*-neglect in existing methods and the complexity of matrix-driven approaches.

### 4.2. Time Series Classification Experiments

#### 4.2.1. Classification Calculation Process

The high dimensionality of time series data provides a robust framework for evaluating classification algorithms. This study employs the UCI Synthetic Control Chart Time Series dataset [21] (6 classes, 600 instances × 60 timestamps), where each class contains 100 homogeneous time series simulating industrial control scenarios.

Data partitioning followed established protocols: the first 90 instances per class formed the training set (540 total), while the remaining 10 instances constituted the test set (60 total). Building upon literature [13] where HECM achieved minimal classification error, we implemented incremental dimensionality reduction (2–30 dimensions) on both sets to benchmark HECM-Plus against HECM. The classification workflow comprised:

- Step (1) Data Preprocessing Stage: The first 90 rows from each category were selected as the training set, and the remaining 10 rows formed the test set, resulting in a training set of 540 time series and a test set of 60 time series.
- Step (2) Segmentation and Dimensionality Reduction: The dimensionality parameter *d* was adjusted within the range of 2 to 30. Each time series (60 timestamps) was divided into *d* non-overlapping equal-length segments. If the total length was not perfectly divisible by *d*, the remainder data points were truncated to ensure equal segment lengths. The mean value of each segment was calculated to achieve dimensionality reduction.
- Step (3) Reverse Cloud Feature Extraction: The reverse cloud algorithm was applied to each dimensional segment to extract digital features of cloud concepts (*Ex*, *En*, and *He* for HECM-Plus; *Ex* and *En* for HECM).
- Step (4) Similarity Calculation: Using HECM-Plus and HECM, the similarity between cloud concepts in the training set and the test set was computed, and multi-dimensional similarity matrices were constructed.
- Step (5) Classification Decision: Based on the 1-nearest neighbor principle, the category with the highest similarity in each dimensional similarity matrix was selected as the classification result.
- Step (6) Performance Evaluation: The classification error rate (ratio of misclassified samples to the total samples) was calculated to evaluate the algorithm’s classification performance and accuracy.

#### 4.2.2. Classification Calculation Results

**Figure 3:**
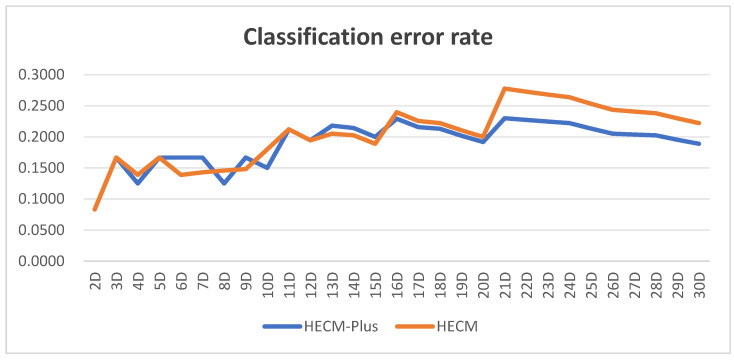
Classification error rate.

Figure 3 shows the classification error rates of HECM-Plus and HECM at different dimensions. Table 4 presents the mean and standard deviation of these error rates. As shown in Figure 3, HECM-Plus exhibits a significantly lower classification error rate than HECM, particularly in high-dimensional settings. As shown in Table 4, HECM-Plus exhibits a lower mean classification error rate and standard deviation than HECM, indicating its superior classification performance and greater stability.

To statistically validate the dimensional superiority observed in Figure 3 and Table 4, a paired t-test was conducted to assess the classification performance of HECM-Plus against HECM. The results yielded a t-statistic of −3.2856 (*p* = 0.0027), indicating a statistically significant difference between the two algorithms at a significance level of α = 0.05. The negative t-value confirms that HECM-Plus achieves a significantly lower mean classification error rate compared to HECM across all dimensional configurations (2D–30D). This finding is consistent with the experimental observations in Figure 3 and Table 4, where HECM-Plus demonstrates reduced error variance and improved robustness in high-dimensional time-series classification tasks.

In summary, HECM-Plus offers several significant advantages over existing methods, including:

- Comprehensiveness and Accuracy: HECM-Plus effectively captures the geometric characteristics of cloud concepts and quantifies their differences by considering three essential numerical features: *Ex*, *En*, and *He*. This method minimizes information loss and significantly improves the accuracy and reliability of concept differentiation and classification. In particular, *Ex* indicates the central tendency of cloud concepts, *En* captures their ambiguity, and *He* represents the degree of their discreteness. By incorporating these features, HECM-Plus effectively captures the core characteristics of cloud concepts, resulting in more dependable classification results for complex datasets. This method improves classification accuracy and enhances the robustness of the model, ensuring consistent and stable performance across various dataset types and sizes.
- Computational Efficiency: HECM-Plus mainly depends on numerical features to perform direct algebraic operations, eliminating the need for complex integration calculations. Compared with traditional algorithms such as ECM, MCM, and PDCM, HECM-Plus offers a more direct and concise calculation process, leading to a significant reduction in computational complexity. This optimization not only reduces computational resource requirements but also significantly improves computation speed. In particular, when handling large-scale datasets, the algorithm substantially reduces the required time, thereby improving operational efficiency.
- Universality and Extensibility: The Hellinger distance is an f-scattering that meets the criteria of distance axiomatization. Thus, it is suitable for use not only with traditional cloud models but also with higher-order normal clouds and high-dimensional cloud models. The broad applicability of the Hellinger distance is one of its key advantages. The flexibility and adaptability of HECM-Plus allow it to address various real-world challenges, making it a crucial tool in various fields.

## 5. Application of HECM-Plus to Conflict Resolution in Design

In design work evaluation, conventional approaches typically depend on subjective assessments from a team of experts, simplifying the evaluation process by calculating an average score to determine the final grade of the work. However, this simplification discards critical uncertainty information inherent in multi-expert systems. To address this, we adopt a cloud model framework, a three-parameter representation (*Ex*, *En*, *He*) that quantifies expected value (*Ex*), fuzziness (*En*), and stochastic dispersion (*He*) of evaluations. While averaging (*Ex*) is effective for highly consistent evaluations, the cloud model reveals two hidden uncertainties: *En* measures ambiguity in criteria interpretation (e.g., “What defines a ‘good’ design?”), and *He* captures randomness in expert opinions (e.g., “Why do ratings vary so widely?”). This dual perspective is essential for resolving conflicts in scattered or controversial cases.

Table 5 presents the design work scores from a university in Chengdu. Using traditional methods, the average scores of Work 1 and Work 2 were 83.5 and 80.3, respectively, both classified as “good” (80–90 range). However, Work 2 exhibits significant uncertainty: its scores span from 61 to 95 (see Table 5), indicating high *En* (ambiguity in quality criteria) and *He* (random divergence among experts). This complexity is invisible to mean-based methods.

To address this, we employ reverse cloud transformation to model dual uncertainties explicitly. Work 1 is represented as (83.5, 4.0106, 0.87467), while Work 2 becomes (80.3, 8.8985, 2.6882). Here, *En* captures the spread of ratings (fuzziness), and *He* quantifies their stochastic dispersion.

Historical rating clouds are defined as follows:

- Excellent: (95, 2, 0.5),
- Good: (85, 4, 1.0),
- Moderate: (75, 6, 1.5),
- Poor: (50, 8, 2.0).

**Figure 4:**
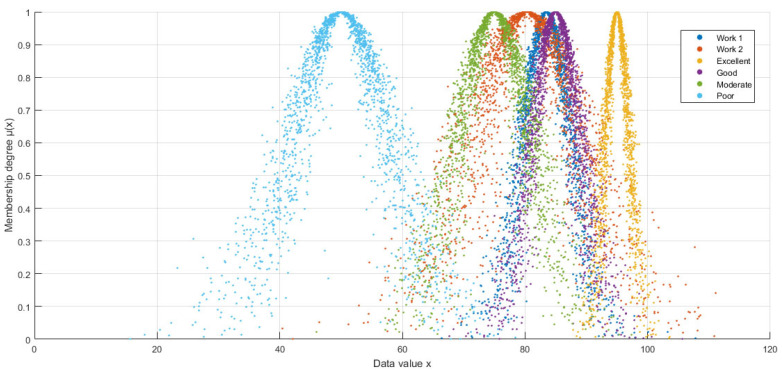
Cloud model representations of design works.

This transformation not only preserves the average rating values but also highlights the ambiguity and randomness of the ratings, providing a more detailed and comprehensive dimension of information for accurate assessment. Figure 4 shows the six clouds, where the deep blue cloud represents the rating cloud of Work 1 and the orange cloud represents the rating cloud of Work 2.

The concentrated morphology of Work 1′s deep blue cloud (lower *En* and He) reflects consistent expert consensus with minimal ambiguity, aligning with the “good” grade’s narrow criteria. In contrast, Work 2′s dispersed orange cloud exhibits greater horizontal spread (*En* = 8.8985) and vertical thickness (*He* = 2.6882), visually quantifying its ambiguous “moderate/good” boundary and stochastic score dispersion (61–95), which structurally overlaps the medium-grade cloud’s probabilistic distribution.

To confirm this observation, HECM-Plus introduced in this study was used for a comparative analysis with HECM. As shown in Table 6, both algorithms consistently categorized Work 1 as “good,” accurately reflecting its rating. This demonstrates the stability and reliability of the algorithms in the evaluation. However, in the more complex evaluation of Work 2, although both algorithms assigned a “moderate” rating, the results differed significantly from those obtained using traditional scoring approaches. In particular, HECM-Plus achieved a similarity of 0.6963 in classifying Work 2 as “moderate,” which is substantially higher than the 0.6157 similarity of HECM. This improvement is attributed to the explicit incorporation of *He* in HECM-Plus, which quantifies the stochastic dispersion of expert opinions. By integrating *He* into the adjusted standard deviation calculation, HECM-Plus captures the inherent randomness in controversial evaluations—such as the scattered ratings of Work 2—thereby enhancing discriminative accuracy. This comparison not only demonstrates that HECM-Plus has higher discriminative accuracy when dealing with complex and scattered data but also further indicates its advantage when dealing with more controversial works.

Moreover, we found that the discriminative margin of HECM-Plus (0.6963 − 0.5713 = 0.125) is substantially higher than that of HECM (0.6157 − 0.5205 = 0.0952) when analyzing the similarity difference between the “medium” and “good” grades. These results further confirm the superiority of HECM-Plus in discriminating adjacent grades and enhancing discrimination accuracy. This improvement stems from HECM-Plus’s explicit modeling of *He*-driven stochastic dispersion, which quantifies the randomness in expert disagreements (e.g., Work 2′s wide score distribution), while *En* captures the inherent ambiguity of qualitative criteria like “moderate” versus “good”. By bridging design studies’ need for aesthetic interpretation with decision theory’s demand for probabilistic rigor, HECM-Plus resolves interdisciplinary conflicts by quantifying both conceptual ambiguity (*En*) and stochastic dispersion (*He*). The method adapts classification boundaries through probability density comparisons when evaluation criteria differ across domains.

In multi-expert design evaluation, the cloud model significantly enhances evaluation efficacy through its certainty (*Ex*), fuzziness (*En*), and randomness (*He*). It quantifies the fuzziness of concepts via *En* (e.g., the semantic boundary dispersion of expressions like “design quality slightly above average”) and characterizes randomness using *He* (e.g., the random fluctuations in different experts’ thresholds for “average”). This mechanism precisely captures the cognitive traits of semantic ambiguity and individual threshold deviations in expert ratings. Furthermore, *Ex* can soften discrete scores (e.g., 80.3 points) into corresponding semantic levels (e.g., “good”) while effectively mitigating the “hard boundary” distortion issue of traditional threshold methods through the constraints of *En* and *He* (e.g., average scores of 79.9 and 80.1 are continuously mapped due to the extensibility of *En* and *He* rather than being forcibly categorized into different levels). The cloud model can transform discrete scores into continuous probability distributions, making it inherently compatible with multidisciplinary experts’ varying interpretations of indicators like “innovativeness”. For example, art experts focus on “aesthetic fuzziness” while mechanical experts emphasize “technical feasibility randomness”. It is precisely because the cloud model characterizes concepts through these three features so that robust aggregation from discrete individual ratings to group consensus can be achieved, providing interpretable support for cross-domain complex evaluations. Therefore, *He* clearly cannot be ignored in the distance measurement of the cloud model. HECM-Plus tackles the issue of traditional cloud models overlooking *He* in distance measurement, offering a scientific and concise algorithm to resolve conflicts in multi-criteria design evaluation while maintaining the probabilistic nature of expert opinions.

## 6. Conclusions

The accurate measurement of cloud model similarity is pivotal for advancing uncertainty-aware decision systems. This study proposed HECM-Plus, a Hyper-entropy-enhanced Hellinger similarity algorithm that integrates *Ex*, *En*, and *He* through reverse cloud transformations. Three key contributions emerge:

- Conceptual discrimination: By reformulating the Hellinger distance with *He*-adjusted standard deviations (Equations (21)–(24)), HECM-Plus achieved a 12.5% higher discriminative margin than HECM in resolving multi-expert conflicts (Table 6), correcting overestimated similarities (e.g., 0.94 vs. 0.99 for *C_1_*–*C_4_* in Table 1) through rigorous uncertainty decomposition.
- Statistical robustness: In time-series classification, HECM-Plus reduced the mean error by 6.8% (0.190 vs. 0.204, *p* < 0.05) and variance by 26% (Table 4), with a paired t-test confirming significant improvement (t = −3.2856, *p* = 0.0027), demonstrating superior stability in high-dimensional settings.
- Conflict resolution in multi-criteria design evaluation (MCDM): By reclassifying controversial designs (e.g., overriding an 80.3 average “good” to “moderate” via *He*-weighted thresholds in Section 5), the algorithm resolved subjective disagreements in expert evaluations, offering a principled framework for design quality assessment.

However, HECM-Plus’s performance depends on the representativeness of expert ratings, where biased or non-random samples may distort *He* estimation. HECM-Plus relies on Gaussian approximations for cloud model distributions, which may degrade performance with heavy-tailed or multi-modal expert evaluation data, as observed in design controversies. Furthermore, the reverse cloud algorithm requires sufficient samples for stable *He* estimation, and limited expert panels may amplify variance in *En*/*He* recovery. Additionally, the current implementation assumes equal expert reliability, meaning biased ratings disproportionately influence *Ex*/*En*/*He* without robustness mechanisms to mitigate such distortions. Biased or non-random samples may affect *He* estimation. Future work will focus on:

- Extending HECM-Plus to higher-order normal clouds and high-dimensional scenarios while maintaining its computational efficiency.
- Integrating reliability metrics to dynamically adjust expert contributions during reverse cloud transformations, mitigating bias in *He* estimation.
- Deploying the algorithm in real-time decision support systems for applications ranging from intelligent design iteration to industrial process monitoring.

These advancements establish HECM-Plus as a theoretically grounded and computationally efficient tool for uncertainty quantification, bridging geometric ambiguity and stochastic dispersion in uncertainty-aware decision analytics.

## Figures and Tables

**Table 1:** Similarity results of different algorithms.

Similarity	LICM	ECM	MCM	PDCM	HECM	HCCM	HECM-Plus
S(C_1_,C_2_)	0.96	0.01	0.33	0.01	0.04	0.22	0.04
S(C_1_,C_3_)	0.97	0.04	0.37	0.03	0.11	0.26	0.08
S(C_1_,C_4_)	0.99	0.94	0.96	0.89	0.99	0.99	0.94
S(C_2_,C_3_)	0.99	0.86	0.95	0.80	0.97	0.86	0.87
S(C_2_,C_4_)	0.97	0.01	0.38	0.01	0.04	0.22	0.04
S(C_3_,C_4_)	0.98	0.04	0.37	0.03	0.11	0.26	0.09

**Table 2:** Variance and standard deviation ratios of HECM vs. HECM-Plus.

Cloud Model	HECM Variance	HECM-Plus Variance	Variance Increase (%)	HECM Standard Deviation Ratio	HECM-Plus Standard Deviation Ratio
*C_1_*	0.3927	0.5076	29.3%	$\sqrt{0.3927/0.3619}\approx 1.042$	$\sqrt{0.5076/0.4571}\approx 1.054$
*C_4_*	0.3619	0.4571	26.3%		

**Table 3:** Degree of variation of different algorithms.

Degree of Variation	LICM	ECM	MCM	PDCM	HECM	HCCM	HECM-Plus
δC_1_	0.05	1.83	1.22	1.74	1.83	1.50	1.76
δC_2_	0.05	1.70	1.19	1.58	1.86	1.28	1.66
δC_3_	0.03	1.64	1.16	1.54	1.72	1.20	1.58
δC_4_	0.03	1.83	1.17	1.74	1.83	1.50	1.76

**Table 4:** Means and standard deviations of classification error rates.

	Mean Classification Error Rate	Standard Deviation of Classification Error Rate
HECM-Plus	0.19033905	0.034925326
HECM	0.204222243	0.047171944

**Table 5:** Expert ratings of design work.

No.	Expert 1	Expert 2	Expert 3	Expert 4	Expert 5	Expert 6	Expert 7	Expert 8	Expert 9	Expert 10
Work 1	88	85	83	82	89	83	88	83	75	79
Work 2	61	90	75	80	85	95	85	82	80	70

**Table 6:** Evaluation results of the cloud models.

Algorithm	Work	Excellent	Good	Moderate	Poor	Grade
HECM-Plus	1	0.0983	0.8716	0.4447	0.0165	Good
	2	0.1989	0.5713	0.6963	0.1199	Moderate
HECM	1	0.0167	0.8144	0.2792	0.0004	Good
	2	0.0936	0.5205	0.6157	0.0204	Moderate

## Data Availability

The original contributions presented in the study are included in the article: further inquiries can be directed to the corresponding author.

## References

[1] Li D.Y., Liu C.Y. (2004). On the universality of normal cloud model. China Eng. Sci..

[2] Mandal S., Khan D.A. (2022). Cloud-CoCoSo: Cloud Model-Based Combined Compromised Solution Model for Trusted Cloud Service Provider Selection. Arab. J. Sci. Eng..

[3] Li W.S., Zhao J., Xiao B. (2018). Multimodal medical image fusion by cloud model theory. Signal Image Video Process.

[4] Liu Y., Liu Z.T., Li S. (2023). Cloud-Cluster: An uncertainty clustering algorithm based on cloud model. Knowl.-Based Syst..

[5] Wu Y.N., Chu H., Xu C.B. (2021). Risk assessment of wind-photovoltaic-hydrogen storage projects using an improved fuzzy synthetic evaluation approach based on cloud model: A case study in China. J. Energy Storage.

[6] Guan J., Liu J., Chen H., Bi W. (2024). A Multi-Criteria Decision-Making Approach for Equipment Evaluation Based on Cloud Model and VIKOR Method. Int. J. Adv. Comput. Sci. Appl..

[7] Chai S.L., Wang Z. (2022). Product design evaluation based on FAHP and cloud model. J. Intell. Fuzzy Syst..

[8] Wang Z., Zhong Y., Chai S.L., Niu S.F., Yang M.L., Wu G.R. (2024). Product design evaluation based on improved CRITIC and Comprehensive Cloud-TOPSIS-Applied to automotive styling design evaluation. Adv. Eng. Inform..

[9] Huang Q., Liu R. (2019). A review of similarity metrics for cloud model. Data Commun..

[10] Zhang G., Li D., Li P., Kang J.C., Chen G.S. (2007). A collaborative filtering recommendation algorithm based on cloud model. J. Softw..

[11] Li H.-L., Gong C.-H., Qian W.-R. (2011). Similarity measurement between normal cloud models. Acta Electron. Sin..

[12] Wang J., Zhu J.J., Liu X.D. (2017). An integrated similarity measure method for normal cloud model based on shape and distance. Sys. Eng.—Theory Pract..

[13] Xu C., Xu H. (2023). Similarity measurement method for normal cloud based on Hellinger distance and its application. CAAI Trans. Intell. Syst..

[14] Li D.Y., Han J.W., Shi X.M., Chan M.C. (1998). Knowledge representation and discovery based on linguistic atoms. Knowl.-Based Syst..

[15] Li D. (1997). Knowledge Representation in KDD Based on Linguistic Atoms. J. Comput. Sci. Technol..

[16] Sun P., Zhang R.Z., Qiu X.W. (2023). A survey on cloud model. J. Internet Technol..

[17] Li Q., Dong Q.K., Zhao L. (2013). Modified forward cloud generator in the cloud model. J. Xidian Univ..

[18] Chen H., Li B., Liu C. (2015). An Algorithm of Backward Cloud without Certainty Degree. J. Chin. Comput. Syst..

[19] Wang G.Y., Xu C.L., Li D.Y. (2014). Generic normal cloud model. Inf. Sci..

[20] Zheng Y., Yang F., Duan J., Kurths J. (2021). Quantifying model uncertainty for the observed non-Gaussian data by the Hellinger distance. Commun. Nonlinear Sci. Numer. Simul..

[21] (1999). Synthetic Control Chart Time Series. http://archive.ics.uci.edu/ml/datasets/Synthetic+Control+Chart+Time+Series.

